# Early feeding practices and stunting in Rwandan children: a cross-sectional study from the 2010 Rwanda demographic and health survey

**DOI:** 10.11604/pamj.2018.29.157.10151

**Published:** 2018-03-19

**Authors:** Etienne Nsereko, Assumpta Mukabutera, Damien Iyakaremye, Yves Didier Umwungerimwiza, Valens Mbarushimana, Manassé Nzayirambaho

**Affiliations:** 1 School of Health Sciences, College of Medicine and Health Sciences, University of Rwanda, Rwanda; 2 School of Public Health, College of Medicine and Health Sciences, University of Rwanda, Rwanda

**Keywords:** Rwanda, feeding practices, childhood stunting, DHS

## Abstract

**Introduction:**

in Rwanda, despite different interventions to improve child nutrition status, malnutrition in children under five years of age continue to be a public health concern. This study aimed to evaluate the factors that contribute to childhood stunting by assessing feeding practices of Rwandans in children ≤ 2 years of age.

**Methods:**

A cross-sectional study with data obtained from the 2010 Rwanda Demographic and Health Survey was conducted on 1,634 children ≤ 2 years of age with complete anthropometrical measurements. Multivariable logistic regression analysis was used to assess the association between feeding practices and childhood stunting.

**Results:**

The results revealed that 35.1% of 1,634 children were stunted. Breastfeeding for 1 year (OR = 2.77, 95% CI = 1.91-4.01, P < 0.001) increased the risk of childhood stunting. After controlling for confounders, solid food initiation (OR = 1.21, 95% CI = 0.47-3.16, P≥ 0.690) and early initiation to breastfeeding (OR = 1.16, CI = 0.90-1.51, P = 0.243) were not associated with childhood stunting.

**Conclusion:**

There was a significant association between continued breastfeeding for 1 year and childhood stunting. We suggest supplementary feeding for children who are breastfed for ≥1 year.

## Introduction

In Rwanda, malnutrition in children under five years of age is a significant public health concern that contributes to infant and child morbidity and mortality [1, 2]. To reduce childhood malnutrition and stunting, the Government of Rwanda has developed a maternal and child nutrition program, “The 1,000 Day Window of Opportunity”, which aims to improve nutrition for both mothers and children from conception to a child's second birthday [3]. In Rwanda, child feeding practices have been documented [4]; however, there is little information on the causes of childhood stunting. Sub-optimal child feeding practices have been associated with stunting in other countries [5]. However, that association has not been observed in Rwanda. The objective of this study was to assess the factors that contribute to childhood stunting by evaluating the feeding practices of Rwandan in children ≤ 2 years of age.

## Method

**Study design:** We obtained data from the 2010 Rwanda Demographic and Health Survey (RDHS) to assess the effects of feeding practices on stunting in children ≤ 2 years of age. The RDHS, which has a cross-sectional design, is performed every five years by the National Institute of Statistics of Rwanda and the Ministry of Health. The 2010 RDHS utilized a two-stage sampling process. In the first stage, 492 villages were randomly selected with a sampling probability proportional to the village size and were stratified by district. In the second stage, 26 households from each village were randomly selected. The response rate was 99.1% from females and 98.7% from males. A total of 8,605 children were included in the survey [**6**]. Anthropometrical measurements were obtained from 4,117 children <5 y of age. A total of 1,634 children were ≤ 2 years of age.

**Figure 1:**
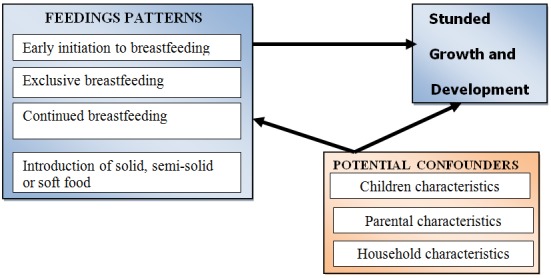
Conceptual framework

**Study variables:** Based on WHO [7] and Rwandan [4] reports, this study hypothesized that feeding practices (i.e., early initiation to breastfeeding; exclusive breastfeeding; continued breastfeeding; and introduction of solid, semi-solid, or soft foods) are independent contributors of stunting in Rwandan children ≤ 2 years of age. Children, parental, and household characteristics were considered to be potential confounders; therefore, they were controlled for in our statistical model (Figure 1).

**Primary outcome:** Our main study outcome was the prevalence of stunting in children ≤ 2 years of age. Anthropometric measurements were converted into nutritional indices [8]. Height-for-age was used to measure childhood stunting; a -2 z-score was indicative of childhood stunting.

**Main predictors: feeding pattern indicators:** The main predictors of interest, which were based on the WHO Infant and Young Child Feeding indicators, included early initiation of breastfeeding; exclusive breastfeeding under 6 months; continued breastfeeding at 1 year; and introduction of solid, semi-solid, or soft foods [9]. Early initiation of breastfeeding represents the number of children born in the previous 24 months who were breastfed within one hour post-childbirth. Early initiation to breastfeeding is considered to be protective due to the health benefits of colostrum in early breast milk. Exclusive breastfeeding under 6 months represents the number of 0 to 5-month-old infants who were exclusively breastfed. Introduction of solid, semi-solid, or soft foods represents the number of 6 to 8-month-old infants who were fed solid, semi-solid, or soft foods [9].

**Potential socio-demographic confounders:** The covariates in this study included children age and sex; maternal age, occupation, and education; family residence (urban vs. rural); number of household members; household family income/wealth index; and accessibility to potable drinking water and adequate sanitation facilities. Wealth index was assessed on the basis of de jure population asset data using principal components analysis. Wealth data were obtained from responses to questions on ownership of certain goods and housing characteristics (e.g., access to electricity and source of drinking water, amongst others) [6].

**Statistical analyses:** Data were analysed using Stata v13 software (StataCorp LP, College Station, TX, USA). Survey commands were used to account for the complex sample design and unequal sampling probability. The associations between feeding practices, socio-demographic variables, and childhood stunting were assessed by simple logistic regression. We tested for confounders using multiple logistic regression. Only variables that modified the coefficient of the outcome by ≥10% were included and controlled for in the final models. Socio-demographic variables that modified the childhood stunting coefficient by ≥10% were included in the multivariable model as potential confounders of the association between childhood stunting and feeding practices. The unadjusted model included childhood stunting and all feeding practice variables. The adjusted model controlled for socio-demographic potential confounders. Odds ratio (OR), 95% confidence interval (CI), and P-values were reported.

**Ethical consideration:** Authorization to use the RDHS dataset was obtained through an online application. The researchers did not make any efforts to identify the survey participants.

## Results

Significantly higher rates of stunting were observed in 18 to 24-month-old children (55.2%), male children (40.4%), children whose mothers were 35 to 49 years of age (43.1%), children whose mothers had no education (42%), and children from low household wealth indices (42.1%). Childhood stunting increased with child age (OR = 6.60, P < 0.001), maternal age (OR = 1.63, P = 0.002), number of household members (OR = 1.38, P = 0.017), residence (OR = 1.82, P = 0.003), source of drinking water (OR = 1.37, P = 0.008), and adequate sanitation facilities (OR = 1.55, P < 0.01). Compared to males, females had lower risk of stunting (OR= 0.63, P < 0.001). On the other hand, childhood stunting was negatively associated with high maternal educational level (OR = 0.27, P < 0.001), high body mass index (BMI; OR = 0.56, P < 0.001), and high household family income (OR = 0.38, P < 0.01; Table 1). Breastfeeding for at least 1 year (OR = 3.72, P < 0.001) and solid food initiation (OR = 1.49, P = 0.01) were associated with childhood stunting (Table 2). There was no significant association between childhood stunting and early initiation to breastfeeding (OR = 0.81, P = 0.090) or exclusive breastfeeding up to 5 months (OR = 0.49, P = 0.641). After adjusting for potential confounders in the multivariable analysis, only continued breastfeeding for at least 1 year was found to be associated with childhood stunting (OR = 2.77, P < 0.001; Table 3). Solid food initiation (OR = 1.21, P = 0.690) and early initiation to breastfeeding (OR = 1.16, P = 0.243) were not associated with childhood stunting after controlling for confounders.

**Table 1:** Distribution and proportion of childhood stunting by socio-demographics characteristics

	N	Number of stunted children(%)	OR (95% CI)	P-value
**Child age (months)**				
< 6	414	65 (15.7)	1.00	
6-12	428	113 (26.4)	1.93 (1.34-2.78)	< 0.001
12-18	393	175 (44.5)	4.29 (3.03-6.08)	< 0.001
18-24	399	220 (55.2)	6.60 (4.60-9.48)	< 0.001
**Child sex**				
Male	803	324 (40.4)	1.00	
Female	831	249 (29.9)	0.63 (0.51-0.77)	< 0.001
**Maternal age (y)**				
15-24	449	143 (31.8)	1.00	
25-34	838	281 (33.5)	1.08 (0.85-1.38)	0.530
35-49	347	150 (43.1)	1.63 (1.19-2.23)	0.002
**Maternal education level**				
No education	285	120 (42)	1.00	
Primary	1,201	429 (35.7)	0.77 (0.58-1.02)	0.069
Secondary and above	148	24 (16.3)	0.27 (0.16-0.45)	< 0.001
**Maternal BMI (not pregnant at time of survey)**				
< 18.5 (underweight)	74	32 (43.1)	1.36 (0.84-2.22)	0.211
18.5-24.9 (normal weight)	1,243	444 (35.7)	1.00	
25.0-29.9 (overweight)	242	57 (23.6)	0.56 (0.40-0.77)	< 0.001
**Nutrition education?**				
No	637	206 (32.4)	1.00	
Yes	994	367 (36.9)	1.22 (0.97-1.53)	0.084
**Number of household members**				
≤ 4	324	95 (29.4)	1.00	
> 4	1,310	478 (36.5)	1.38 (1.06-1.79)	0.017
**Household wealth index**				
Poorest	366	151 (41.2)	1.00	
Poorer	384	161 (42.1)	1.04 (0.78-1.38)	0.812
Middle	323	121 (37.3)	0.85 (0.62-1.17)	0.312
Rich	295	84 (28.6)	0.57 (0.41-0.80)	0.001
Richest	267	56 (21.0)	0.38 (0.26-0.55)	< 0.001
**Residence**				
Urban	184	44 (24.0)	1.00	
Rural	1,450	529 (36.5)	1.82 (1.22-2.71)	0.003
**Source of drinking water**				
Unimproved	465	188 (40.3)	1.00	
Improved	1148	378(33.0)	1.37 (1.09-1.74)	0.008
**Sanitation facilities**				
Inadequate	442	188 (42.4)	1.00	
Adequate	1169	377(32.2)	1.55 (1.24-1.94)	< 0.001
**Total**	1,634	573 (35.1)	-	-

**Table 2:** Distribution of childhood stunting by feeding pattern

Variables	n	Stunted (%)	OR	95% CI	P-value
**Continued breastfeeding (12-15 months)**					
< 1 y	748	146 (19.6)	1.00	1.00	
1 y	242	98 (40.6)	3.72	(2.88-4.80)	< 0.001
**Early initiation to breastfeeding**					
≥ 1 h post-childbirth	438	165 (37.6)	1.00	1.00	
< 1 h post-childbirth	1140	375 (32.9)	0.81	(0.64-1.03)	0.090
**Solid food initiation?**					
No	510	89 (17.4)	1.00	1.00	
Yes	45	9 (20.5)	1.49	(1.19-1.86)	0.010
**Exclusive breastfeeding at 5 months**					
No	54	10 (19.1)	1.00	1.00	
Yes	300	49 (16.3)	0.49	(0.38-0.63)	0.641

**Table 3:** Multiple logistic regression models for each feeding practices and childhood stunting

Feeding practices	Unadjusted OR^†^	95% CI	P-value	Adjusted OR*	95% CI	P-value
**Continued breastfeeding**						
< 1 y	1.00			1.00		
1 y	2.81	(1.96-4.03)	< 0.001	2.77	(1.91-4.01)	< 0.001
**Early initiation to breastfeeding**						
≥ 1 h post-childbirth	1.00			1.00		
< 1 h post-childbirth	1.23	(0.97-1.57)	0.090	1.16	(0.90-1.51)	0.243
**Solid food initiation?**						
No	1.00			1.00		
Yes	1.23	(0.48-3.14)	0.671	1.21	(0.47-3.16)	0.690
**Constant**	0.24	(0.20-0.29)	< 0.001	0.27	(0.11-0.67)	0.005

## Discussion

This study assessed the association between feeding practices and stunting in children ≤ 2 y of age. Among the feeding patterns, breastfeeding for >1 y was a risk factor of childhood stunting even after controlling for confounders; similar results have been previously reported [10, 11]. A study performed with Peruvian kids revealed an inverse relationship between extended breastfeeding (i.e., for more than one year) and late childhood stunting [10]. Several studies have explored the relationship between prolonged breastfeeding and childhood stunting [12, 13]. It has been postulated that prolonged breastfeeding is a response to poor growth [12], and that poor linear growth is a consequence of inadequate nutrients intake [14]. Prolonged breastfeeding increases the risk of nutrient deficiencies [11]. Rwanda has made considerable efforts to develop and implement complementary feeding practices and guidelines through its “The 1,000 Day Window of Opportunity” program. However, there are no reports on the effectiveness of these feeding practices. The results of our study revealed that breastfeeding for >1 y delayed the introduction of complementary foods, as reported by the Knowledge, Attitude, and Practice (KAP) study [4]. This study did not found any association between early initiation to breastfeeding or exclusive breastfeeding and childhood stunting. Our results agree with the findings obtained by Kramer and Kakuma, who reported no associations between exclusive breastfeeding and growth [15]. Childhood stunting is less prevalent among infants <6 months of age. The prevalence of childhood stunting increases with age. Our findings suggest that any protective effect conferred by exclusive breastfeeding does not overcome the adverse factors that affect children: poor nutrient intakes, infections, and diarrhea [15].

This study had some limitations. First, it was a cross-sectional survey based on 24 hours recalls, which are subject to recall biases. Second, the RDHS provided information on milk consumption using a “yes/no” format. Therefore, it was not possible to examine all WHO indicators, particularly diet quality and consumption frequency in children 6 to 23 months of age.

**Strengths and limitations of this study:** 1) this study used a representative sample to investigate the association between feeding practices and childhood stunting; 2) however, a cross-sectional study cannot determine cause and effect; 3) the study did not examine all WHO indicators, particularly diet quality and consumption frequency in children 6 to 23 months of age.

## Conclusion

Breastfeeding for >1 year was associated with childhood stunting. Further research is required to explore the relationship between childhood stunting and prolonged periods of breastfeeding. The maternal and child nutrition program, “The 1,000 Day Window of Opportunity”, should focus on feeding practices and supplementary feeding for children who are breastfed for 1 year or more.

### What is known about this topic

- Child feeding practices in Rwanda;
- Sub-optimal child feeding practices have been associated with stunting in other countries.

### What this study adds

- Continued breastfeeding for at least 1 year was found to be associated with childhood stunting;
- Solid food initiation and early initiation to breastfeeding were not associated with childhood stunting.

## Competing interests

The authors declare no competing interests.
